# Integrated Analysis of Zinc, Copper, and Magnesium Homeostasis in Pediatric Idiopathic Nephrotic Syndrome: A Prospective Cohort Study with Serial Clinical Evaluation

**DOI:** 10.3390/nu18152529

**Published:** 2026-08-04

**Authors:** Elena Jechel, Emil Anton, Mitica Ciorpac, Iuliana Magdalena Starcea, Catalina Lunca, Ancuta Lupu, Adriana Mocanu, Sorana Caterina Anton, Anca Adam Raileanu, Otilia Elena Frasinariu, Oana Raluca Temneanu, Ruxandra Russu, Alin Horatiu Nedelcu, Elena Cristina Mitrofan, Vasile Valeriu Lupu

**Affiliations:** 1 Grigore T. Popa University of Medicine and Pharmacy, 700115 Iasi, Romania; elena.jechel@yahoo.com (E.J.); emil.anton@umfiasi.ro (E.A.); mitica.ciorpac@umfiasi.ro (M.C.); catalina.lunca@umfiasi.ro (C.L.); maria-adriana.mocanu@umfiasi.ro (A.M.); sorana.anton@umfiasi.ro (S.C.A.); anca.adam-raileanu@umfiasi.ro (A.A.R.); frasinariu.otilia@umfiasi.ro (O.E.F.); temneanu.oana@umfiasi.ro (O.R.T.); alin.nedelcu@umfiasi.ro (A.H.N.); vasile.lupu@umfiasi.ro (V.V.L.); 2 Faculty of Medicine, “George Emil Palade” University of Medicine, Pharmacy, Science and Technology, 540142 Targu Mures, Romania; ruxandra_rss@yahoo.com; 3 CF Clinical Hospital, 700506 Iasi, Romania; criselend@yahoo.com

**Keywords:** pediatric nephrotic syndrome, trace elements, zinc, copper, magnesium, proteinuria, disease activity, biomarkers

## Abstract

**Background**: Idiopathic nephrotic syndrome (NS) in children is characterized by urinary protein loss and potential disruptions in trace element homeostasis. The dynamic changes in zinc, copper, and magnesium levels in relation to disease activity remain incompletely defined. **Objective**: This study aimed to evaluate serum zinc, copper, and magnesium and urinary copper and magnesium alterations in homeostasis in pediatric nephrotic syndrome and to examine their associations with disease stage, proteinuria, disease duration, renal function, and corticosteroid response. **Materials and Methods:** This is a prospective cohort study involving 108 participants, including 74 pediatric patients with idiopathic nephrotic syndrome and 34 healthy controls, comprising 164 clinical and biological assessments. Serum and urinary concentrations of Zn, Cu, and Mg were analyzed, alongside clearance parameters and the fractional excretion of magnesium. Statistical analysis included non-parametric tests, Spearman correlations, ROC analysis, and multivariable logistic regression. **Results**: Serum zinc levels were significantly lower during active disease phases and normalized during remission (*p* < 0.001); however, these differences in serum zinc concentration with stages of the NS disappeared after adjustment for serum protein levels (*p* = 0.424), suggesting a transport deficit secondary to hypoproteinemia. Although serum zinc concentrations were also significantly reduced in patients with concomitant infection, adjustment for serum protein levels attenuated this association, and the zinc-to-protein ratio did not differ significantly according to infection status (*p* = 0.08). Urinary copper levels and clearance were elevated during active disease and positively correlated with proteinuria (rho = 0.35–0.38; *p* < 0.001); the Cu/Zn ratio varied significantly across disease stages (*p* < 0.001) and was associated with both disease activity and a tendency toward corticosteroid resistance. Magnesium demonstrated a pattern of tubular conservation during active phases, with elevated fractional excretion values during remission (*p* < 0.001) and inverse correlations with proteinuria. Disease duration, but not relapse burden, was positively correlated with serum zinc and fractional magnesium excretion and inversely correlated with serum magnesium. In the multivariable analysis, serum proteins emerged as the sole independent predictor of disease activity, while age and the Cu/Zn ratio were associated with corticosteroid resistance. **Conclusions**: Pediatric nephrotic syndrome induces significant alterations in zinc, copper, and magnesium homeostasis, dependent on glomerular permeability and plasma protein status. Zinc changes with stages of NS likely reflect a secondary transport defect. In contrast, zinc changes with infection likely occurred because of a shift of zinc to the intracellular compartment. Urinary copper serves as a marker of glomerular permeability and magnesium highlights tubular adaptation. The Cu/Zn ratio and magnesium handling parameters may prove clinically useful in monitoring disease activity and treatment response.

## 1. Introduction

Idiopathic nephrotic syndrome (NS) is the most common glomerulopathy in children, with an estimated incidence of 2–7 cases per 100,000 children annually and a predominance of the steroid-sensitive form in the Caucasian population. It is characterized by nephrotic-range proteinuria, hypoalbuminemia, edema, and hyperlipidemia, and typically follows a relapsing course with alternating remissions and relapses [1,2,3]. Although corticosteroid-sensitive forms generally have a favorable prognosis, a substantial proportion of patients develop frequent relapses or steroid dependence, while a smaller subset presents steroid resistance—either at onset or during disease evolution—which is associated with an increased risk of progression to chronic kidney disease [4,5].

Proteinuria, the hallmark of NS, results from disruption of the glomerular filtration barrier and leads not only to urinary albumin loss but also to disturbances in the homeostasis of circulating micronutrients [6,7,8,9]. Approximately 80% of plasma zinc is bound to albumin, copper circulates predominantly bound to ceruloplasmin with a smaller albumin-bound fraction, and 20–30% of circulating magnesium is protein-bound, mainly to albumin. Consequently, urinary loss of carrier proteins may alter the circulating concentrations of these elements independently of nutritional intake [10,11,12,13].

Both classic and contemporary studies have consistently demonstrated reduced serum zinc concentrations during active NS, accompanied by increased urinary zinc excretion proportional to the degree of proteinuria, supporting proteinuria-dependent renal loss as the principal mechanism of hypozincemia [14,15,16,17,18,19,20]. Similarly, urinary copper excretion and clearance increase during active disease and correlate with proteinuria severity. Because copper circulates predominantly bound to ceruloplasmin, changes in serum copper may reflect both proteinuria-related homeostatic disturbances and systemic inflammation. Although the serum copper/zinc (Cu/Zn) ratio has been proposed as an integrative biomarker of inflammation and oxidative stress, evidence in pediatric NS remains limited, and its prognostic value requires further validation [14,20,21,22].

Compared with zinc and copper, magnesium has been less extensively investigated. Available studies suggest lower serum magnesium concentrations during active disease, associated with disease activity and glomerular dysfunction, while calcineurin inhibitor therapy may further increase magnesium loss through effects on tubular TRPM6 transporters [23,24,25]. However, whether these abnormalities represent true micronutrient deficiency or redistribution secondary to plasma protein loss remains uncertain, particularly for zinc and copper, whose circulating concentrations largely depend on protein-bound transport. This distinction has important implications for supplementation strategies, whose clinical benefit remains unproven [26].

Despite these observations, current evidence is limited by predominantly cross-sectional studies involving small cohorts, inadequate stratification according to disease stage (onset, relapse, and early, late, or sustained remission), and the lack of integrated analyses combining serum and urinary micronutrient measurements with renal handling indices and plasma protein status. Consequently, the relative contribution of renal loss and redistribution remains unclear. Moreover, emerging evidence suggests the existence of distinct biological NS subphenotypes characterized by different profiles of oxidative stress, subclinical inflammation, and micronutrient imbalance [27,28,29,30].

To address these knowledge gaps, the present study comprehensively evaluated zinc, copper, and magnesium homeostasis across the clinical spectrum of pediatric idiopathic NS by integrating serum and urinary measurements, renal handling parameters, and their associations with proteinuria, renal function, disease activity, and corticosteroid response. This approach aimed to improve the understanding of micronutrient alterations in pediatric NS and to explore their potential utility as biomarkers of disease activity and therapeutic risk stratification.

## 2. Materials and Methods

### 2.1. Study Design

This was a prospective observational cohort study conducted to evaluate changes in zinc, copper, and magnesium homeostasis during the clinical course of idiopathic NS in children. Patients were monitored through repeated clinical and laboratory assessments performed at various stages of the disease, enabling the characterization of temporal variations in trace element metabolism in relation to disease activity, proteinuria severity, renal function, and treatment response. The primary outcome of the study was the comparison of serum zinc, copper, and magnesium concentrations, together with urinary copper and magnesium excretion, across the different clinical stages of pediatric nephrotic syndrome and to analyze their relationship with clinical and biological parameters of disease activity. All additional correlation, ROC, subgroup, and regression analyses were considered exploratory and hypothesis-generating.

Secondary objectives included evaluating associations between trace element homeostasis and proteinuria, inflammatory markers, renal function, long-term clinical course, and response to corticosteroid therapy, as well as analyzing their potential value as biomarkers of disease activity and resistance to corticosteroid treatment.

### 2.2. Study Population

The study included pediatric patients with idiopathic NS who were under the care of the Pediatric Nephrology Clinic at the “Santa Maria” Children’s Clinical Hospital in Iași—a regional referral center for northeastern Romania. The study enrolled all children diagnosed with NS at onset or those who had at least one presentation for emergency or elective evaluation between February 2024 and February 2025. Additionally, a control group of 34 healthy children—matched for age and sex, and with no relevant medical history—was enrolled. Clinical and biological data were collected prospectively during routine consultations, in accordance with standard monitoring practices.

The final analysis included 164 clinical and laboratory evaluations from 108 unique patients. Based on their clinical status at the time of evaluation, patients with NS were classified into three operational groups: new-onset NS, chronic active NS, and chronic inactive NS (absence of relapse for >12 months).

To analyze disease dynamics, the assessments were subsequently stratified into six clinical stages: initial presentation at diagnosis, initial remission following onset, relapse, early remission (first documented remission), late remission (spaced remission), and sustained remission exceeding 12 months.

Inclusion criteria targeted children under the age of 18 with a diagnosis of idiopathic NS established according to international KDIGO and IPNA guidelines—whether at disease onset or during clinical follow-up—provided that complete clinical and laboratory data were available. The control group consisted of children with no known renal pathology or chronic inflammatory or metabolic diseases, and whose clinical assessments and laboratory results were within normal limits [1,2,3].

Patients with congenital or secondary NS, stage 3 or more advanced chronic kidney disease, urinary tract malformations, genetic renal disorders, active autoimmune diseases, neoplasms, or severe liver disease, as well as those with incomplete physiological and pathological medical histories, were excluded. Children receiving trace element supplements prior to biological sample collection were also excluded.

### 2.3. Clinical Assessment and Laboratory Determinations

Demographic, anthropometric, clinical, and biological data were recorded for each clinical assessment. Clinical parameters included age, sex, body weight, height, body mass index, disease duration, number of relapses, type of response to corticosteroid therapy (sensitive, dependent, or resistant), clinical form of NS (pure or impure), presence of concomitant infections, and administered immunomodulatory treatments.

Venous blood samples were collected in the morning under standardized conditions. For patients at the time of diagnosis or during a relapse, samples were collected prior to the initiation of curative treatment or as close as possible to the onset of the acute episode. Laboratory analyses included a complete blood count, total serum proteins, albumin, creatinine, urea, electrolytes, a complete lipid profile, erythrocyte sedimentation rate, C-reactive protein, and estimated glomerular filtration rate. Concurrently, 24-h urine samples were collected to determine urine volume, proteinuria, and urinary creatinine.

Blood samples intended for trace element analysis were collected using trace-element collection tubes. After routine biochemical analyses, the remaining serum was immediately separated by centrifugation, aliquoted into trace-element-free microtubes, and stored at temperatures between −20 °C and −80 °C, according to the manufacturer’s recommendations for the analytical reagents, until batch analysis. Twenty-four-hour urine samples were aliquoted and stored under identical conditions.

At the end of the study enrollment period, these samples were used to measure serum zinc, copper, and magnesium levels, as well as urinary copper and magnesium excretion. Only total serum zinc, copper, and magnesium concentrations were determined. Urinary copper and urinary magnesium were measured, whereas urinary zinc (zincuria) could not be assessed because of technical limitations. Free (unbound) fractions of zinc, copper, and magnesium were not measured. Serum zinc, copper, and magnesium concentrations, as well as urinary copper and magnesium concentrations, were determined using validated colorimetric assays on an Atellica CH automated chemistry analyzer (Siemens Healthineers, Erlangen, Germany). Zinc and copper were measured using Sentinel Diagnostics reagents (Sentinel Diagnostics, Milan, Italy), whereas magnesium was measured using Siemens Healthineers reagents, according to the manufacturers’ instructions in the hospital’s clinical laboratory.

Based on these measurements, parameters of renal handling were calculated, including copper clearance, magnesium clearance, the fractional excretion of magnesium, and the serum copper/zinc ratio. To account for the influence of hypoproteinemia on trace element distribution, ratios adjusted for total serum protein were also calculated for zinc, copper, and magnesium.

### 2.4. Definitions

Disease activity was defined based on the clinical status of the NS at the time of each assessment, using an operational classification comprising six stages of progression. Initial presentation was defined as the first documented episode of NS, characterized by nephrotic-range proteinuria, hypoalbuminemia, and clinical edema. Relapse was defined as the recurrence of nephrotic-range proteinuria following the achievement of complete remission. Initial remission was defined as the disappearance of nephrotic-range proteinuria within one month of onset or the initiation of treatment. Early remission was defined as the maintenance of remission for a period of one to two months, whereas late remission was defined as the stabilization of the clinical response for a duration exceeding two months. Sustained remission was defined as the absence of relapses for a period exceeding 12 months under continuous clinical monitoring [1,2].

Response to corticosteroid therapy was classified according to current IPNA and KDIGO criteria into: steroid-sensitive NS (defined by achieving complete remission under standard corticosteroid treatment); steroid-dependent NS (defined by the occurrence of relapses during dose reduction or shortly after discontinuing therapy); and steroid-resistant NS (defined by the failure to achieve remission after an adequate course of corticosteroid therapy according to guidelines) [1,2,3].

The estimated glomerular filtration rate (eGFR) was calculated using the updated Schwartz formula—based on serum creatinine, body length, and a constant (0.413)—in accordance with current nephrological recommendations for the pediatric population [31].

Renal handling parameters for the trace elements were calculated using standard formulas based on serum and urinary concentrations determined from simultaneous blood and 24-h urine samples. Copper and magnesium clearances were calculated using the classic renal clearance formula, incorporating the urinary and serum concentrations of the element in question and the urine flow rate. The fractional excretion of magnesium (FEMg) was calculated based on urinary and serum magnesium and creatinine concentrations and expressed as a percentage. Since the study aimed to compare FEMg values across different stages of NS rather than classify patients based on diagnostic thresholds, the same calculation formula was applied consistently to all measurements, without including the 0.7 correction factor. This approach was chosen to avoid assuming a constant ultrafilterable fraction of plasma magnesium, given that magnesium protein binding and its ultrafilterable fraction can vary under conditions associated with alterations in plasma proteins, including hypoalbuminemia [32,33,34,35].

Additionally, the serum copper/zinc (Cu/Zn) ratio was used as an integrative marker of trace element homeostasis; to control for the confounding effect of hypoproteinemia, ratios adjusted for total serum proteins were calculated for zinc, copper, and magnesium, allowing for the differentiation between plasma losses and actual losses of distribution proteins.

### 2.5. Ethical Considerations

The study was approved by the Ethics Committee of the “Grigore T. Popa” University of Medicine and Pharmacy in Iași (499/30 November 2024), as well as by the Ethics Committee of the “Santa Maria” Children’s Clinical Hospital in Iași (No. 38793/20 November 2024). Written informed consent was obtained from the parents or legal guardians of all participants, and the children’s assent was obtained whenever possible, in accordance with the principles of the Declaration of Helsinki.

### 2.6. Statistical Analysis

Statistical analysis was performed using R software (version 3.4; R Foundation for Statistical Computing, Vienna, Austria). Some participants contributed more than one clinical assessment during follow-up, reflecting different stages of disease evolution (onset, relapse, and remission). These repeated observations represented distinct clinical episodes rather than duplicate laboratory measurements. Because the objective was to describe trace element profiles across clinically defined disease stages, each clinical assessment was analyzed as an independent observational time point. This approach should be considered exploratory and represents a limitation of the study.

Continuous variables were described using the median and interquartile range or, where appropriate, the mean and standard deviation, depending on the data distribution. As the distributions were non-normal, only non-parametric tests were used. Comparisons of variables between clinical groups (control, NS onset, chronic active disease, and chronic inactive disease) and between types of clinical episodes (initial presentation, relapse, initial remission, early remission, late remission, and sustained remission) were performed using the Kruskal–Wallis test, followed by pairwise comparisons where appropriate.

Categorical variables were expressed as absolute frequencies and percentages. Hematological, biochemical, inflammatory, urinary, and trace element parameters were analyzed consistently using non-parametric methods. Correlations between variables were assessed using the Spearman coefficient (rho), and comparisons between independent subgroups (presence versus absence of concomitant infection) were performed using the Mann–Whitney U test.

Ratios adjusted for serum proteins (zinc/protein, copper/protein, and magnesium/protein) were compared across groups using the Kruskal–Wallis test. The diagnostic performance of biomarkers was evaluated via ROC curve analysis using the area under the curve (AUC), with sensitivity and specificity reported for the analyzed thresholds. Before fitting the multivariable logistic regression models, multicollinearity among candidate predictors was assessed using variance inflation factors (VIF). All VIF values were below 3 (maximum VIF = 2.59), indicating no evidence of relevant multicollinearity. Therefore, all predefined predictors were retained in the final models. Multivariable logistic regression models were constructed to identify independent predictors of disease activity and corticosteroid resistance, with results expressed as odds ratios with 95% confidence intervals.

Overall, a statistical significance threshold of *p* < 0.05 was adopted for all analyses. Because this was an exploratory prospective observational study, no formal a priori sample size or power calculation was performed. All eligible patients during the study period were included. Likewise, *p*-values were not adjusted for multiple comparisons, and all secondary analyses should be interpreted as exploratory and hypothesis-generating.

## 3. Results

### 3.1. Demographic and Baseline Clinical Characteristics

The final dataset comprised 164 study observations obtained from 108 unique participants, including 74 children with idiopathic nephrotic syndrome who contributed 130 longitudinal clinical evaluations and 34 healthy controls who each contributed one baseline evaluation. Based on current clinical presentation and dynamic disease tracking, the evaluations were stratified into four distinct operational study groups: Group A (Healthy Controls; N = 34, 20.73%), Group B (New-Onset Nephrotic NS; N = 30, 18.29%), Group C (Chronic Active NS; N = 83, 50.61%), and Group D (Chronic Inactive NS; N = 17, 10.37%). The median age of the overall cohort at presentation was 86.5 months (IQR: 53.5–141.25), with a predominant male distribution (N = 104, 63.41%). Group B (New-Onset NS) presented at a significantly younger age (Median: 41.5 months, IQR: 31.2–63.5) compared to Group D (Chronic Inactive NS; Median: 122 months, IQR: 86–148). Anthropometric differences across the study groups were consistent with the observed age distribution. As expected, children with new-onset nephrotic syndrome had lower mean body weight and height than those with chronic inactive disease, while BMI was comparable across all groups. These data are summarized in Table 1. Detailed baseline characteristics, clinical phenotypes (pure vs. impure syndromes), concomitant infections, and ongoing therapeutic regimens (corticosteroids, calcineurin inhibitors, or mycophenolate mofetil) across the four functional strata are comprehensively summarized in Table 2.

### 3.2. Laboratory and Urinary Kinetics Across NS Episode Types

To capture the exact biological shifts during different phases of the disease, the 130 nephrotic evaluations were classified chronologically into six distinct episode types: initial presentation (N = 11), initial remission (remission <1 month; N = 9), relapse (N = 28), early remission (remission 1–2 months; N = 22), late remission (>2 months; N = 43), and sustained remission (remission >12 months; N = 17).

#### 3.2.1. Hematological Parameters

Hematological parameters remained largely stable across disease phases. However, Hematocrit (Hct) values demonstrated statistically significant dynamic variations (*p* = 0.04, Kruskal–Wallis), peaking during the precocious remission phase (Median: 40.45%, IQR: 38.35–42.10%). Pairwise post-hoc analysis via Dunn’s test isolated highly significant differences between early remission and late remission (*p* = 0.001), as well as significant variations between initial presentation vs. early remission (*p* = 0.03) and relapse vs. early remission (*p* = 0.04), tracking intravascular fluid shifts. Complete hematological parameters are detailed in Supplementary Table S1.

#### 3.2.2. Biochemical and Inflammatory Profiles

As expected, active nephrotic syndrome was characterized by marked hypoalbuminemia, hypoproteinemia, hyperlipidemia, increased ESR and nephrotic-range proteinuria, all of which normalized during remission (Table 3). While C-reactive Protein (CRP) remained low and unvaried across all phases (*p* = 0.63), establishing a non-inflammatory mechanism for ESR acceleration. Glomerular hyperfiltration was evident at initial presentation (eGFR Median: 229.73 mL/min/1.73 m^2^) and gradually stabilized back to physiological ranges in late remission (*p* < 0.001). Complete biochemical and inflammatory profiles are detailed in Table 3 and Supplementary Table S1.

#### 3.2.3. Urinary Metrics

Urinary excretion patterns strongly reflected the disruption of the glomerular filtration barrier (Table 3, Supplementary Table S1). Notably, 24-h proteinuria was massive during active disease states (initial presentation: Median 3761.88 mg/24-h; relapse: Median 3704.48 mg/24-h) and plummeted immediately upon reaching early remission (Median 124.08 mg/24-h, *p* < 0.001). This was accompanied by a relative oliguria during initial presentation (24-h Urine Volume Median: 540.00 mL/24-h) which expanded significantly as patients achieved a stable remission state (Median: 1200.00 mL/24-h, *p* = 0.021).

### 3.3. Serum and Urinary Microelement Kinetics

**Figure 1:**
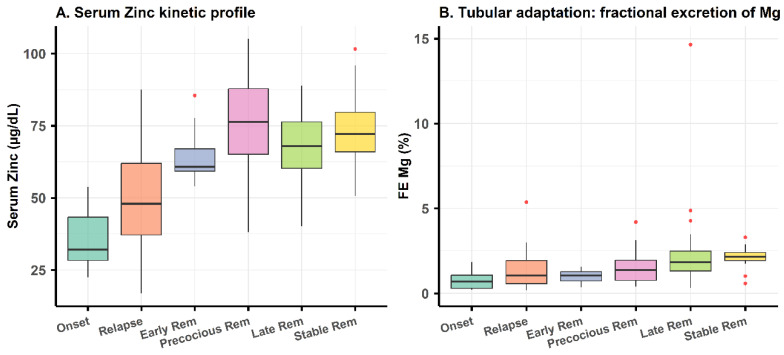
Dynamic kinetic profiles of serum zinc and fractional excretion of magnesium across nephrotic syndrome clinical episodes. (**A**) Boxplot illustrating the profound drop in serum zinc levels during active phases (Onset [Median: 32.10 μg/dL] and Relapse [Median: 48.00 μg/dL]), followed by a progressive, statistically significant normalization throughout early, late, and sustained remission phases (*p* < 0.001). (**B**) Boxplot showing the fractional excretion of magnesium across the same chronological episodes. The minimal excretion noted during Onset (Median: 0.70%) and Relapse reveals a powerful compensatory tubular reabsorption mechanism during heavy proteinuric states, which gradually transitions back to standard baseline excretion in sustained remission (*p* < 0.001). Red markers indicate statistical outliers detected within the sub-cohorts. For graphical display, values exceeding 15% were capped at 15% (N = 2) to improve visualization. All analyses were performed using the original, unmodified values. Abbreviations: Rem = remission.

The core objective of the study revealed substantial homeostatic alterations in zinc, copper, and magnesium handling across disease phases (Table 3, Supplementary Table S1). Serum zinc concentrations were lowest during initial presentation (Median: 32.10 µg/dL) and relapse (Median: 48.00 µg/dL), followed by a steady restoration up to normal levels in late remission (Median: 68.00 µg/dL, *p* < 0.001). Conversely, active states were characterized by hypercupriuria. Both 24-h urinary copper excretion and copper clearance were elevated at initial presentation (73.35 µg/24-h and 0.06 mL/min, respectively) and relapse (84.22 µg/24-h and 0.06 mL/min), dropping down to zero in stable remission (*p* < 0.001). The systemic copper/zinc ratio proved highly responsive, shifting from a peak of 1.90 at initial presentation down to a physiological baseline of 1.24 in stable remission (*p* < 0.001). Serum magnesium dipped slightly during active phases (*p* = 0.006). Notably, the kidney demonstrated an intense tubular conservation pattern during active proteinuric phases: 24-h urinary magnesium excretion, magnesium clearance, and the fractional excretion of magnesium were minimal at initial presentation (20.60 mg/24-h, 0.81 mL/min, and 0.70%, respectively) and rose significantly during stable remission states (Median: 2.17%, *p* < 0.001). These dynamic changes in serum zinc concentrations and fractional magnesium excretion across the different clinical episodes are illustrated in Figure 1.

### 3.4. Correlation Matrices (Proteinuria, Total Proteins, Renal Function, and Inflammation)

To explore the underlying biological associations, Spearman rank correlation (rho) matrices were calculated across the entire database:

In this study, 24-h proteinuria correlated significantly and inversely with serum zinc (rho = −0.38, *p* < 0.001) and the fractional excretion of magnesium (rho = −0.39, *p* < 0.001). It correlated directly and positively with urinary copper concentrations (rho = +0.35, *p* < 0.001) and copper clearance (rho = +0.38, *p* < 0.001), confirming a passive, protein-dependent urinary spilling mechanism for copper (Table 4).

Total serum proteins showed a moderate-to-strong positive correlation with serum zinc (rho = +0.59, *p* < 0.001) and a weak-to-moderate correlation with the Cu/Zn ratio (rho = −0.38, *p* < 0.001). A weak but statistically significant direct correlation was also noted with serum magnesium (rho = +0.16, *p* = 0.04), whereas no significant linear connection was tracked with serum copper (rho = +0.13, *p* = 0.11, Supplementary Table S2).

eGFR displayed a significant, weak-to-moderate inverse correlation with serum zinc (rho = −0.40, *p* < 0.001) and the fractional excretion of magnesium (rho = −0.37, *p* < 0.001). A weak but statistically significant inverse relationship was also observed between eGFR and serum magnesium (rho = −0.19, *p* = 0.02). In contrast, eGFR did not demonstrate significant linear trends with magnesium clearance (rho = −0.14, *p* = 0.08) or serum copper (rho = +0.02, *p* = 0.79, Supplementary Table S3). The systemic Cu/Zn ratio demonstrated a highly significant, moderate positive correlation with ESR (rho = +0.44, *p* < 0.001) and a weak positive correlation with CRP (rho = +0.25, *p* = 0.002). Serum zinc tracked a significant inverse relationship with ESR (rho = −0.32, *p* = 0.001) but did not establish a significant correlation with CRP levels (rho = −0.12, *p* = 0.14). Additionally, serum copper displayed a weak but direct link to CRP (rho = +0.23, *p* = 0.004), while failing to cross the significance threshold for ESR (rho = +0.18, *p* = 0.07, Table 5).

**Figure 2:**
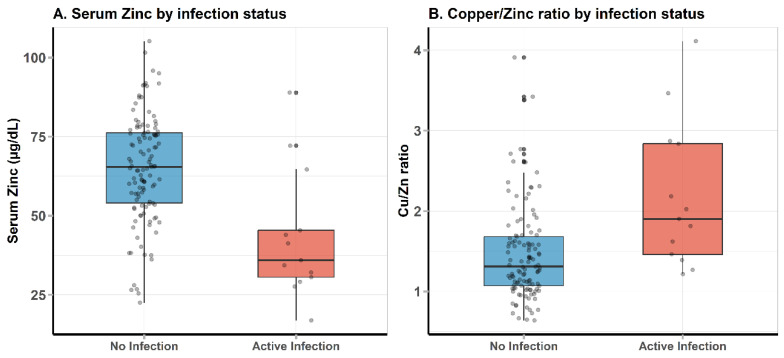
Impact of active concomitant infections on systemic zinc and copper/zinc homeostasis. (**A**) Grouped boxplot combined with jittered individual data points demonstrating a severe, highly significant depletion of serum zinc in pediatric patients presenting with a concurrent active infection (Median: 35.90 μg/dL, IQR: 30.60–45.40) compared to infection-free patients (Median: 65.40 μg/dL, IQR: 54.00–76.20, *p* < 0.001 via Mann–Whitney U test). (**B**) Grouped boxplot illustrating the syndromic amplification and explosion of the systemic copper/zinc ratio driven by infectious status, shifting from a baseline median of 1.31 up to an elevated median of 1.90 in the infected cohort (*p* = 0.001), highlighting a dual-pathological mechanism combining mechanical urinary wasting with cytokine-mediated intracellular sequestration.

Stratifying patients by the presence (N = 13) or absence (N = 117) of an active concomitant infection using the Mann–Whitney U test unveiled highly significant interactions regarding zinc homeostasis metrics. Patients presenting with an active concurrent infection demonstrated heavily suppressed serum zinc levels (Median: 35.90 μg/dL, IQR: 30.60–45.40) compared to those without infections (Median: 65.40 μg/dL, IQR: 54.00–76.20, *p* < 0.001). After normalization for total serum proteins, the zinc-to-protein ratio showed only a non-significant trend toward lower values in patients with concomitant infection compared with those without infection (median 0.93 vs. 1.02, *p* = 0.08). Consequently, the Cu/Zn ratio was significantly elevated in the infection-positive cohort (Median: 1.90, IQR: 1.46–2.84) relative to the infection-negative group (Median: 1.31, IQR: 1.07–1.68, *p* = 0.001). No significant infectious interactions were noted for serum copper (*p* = 0.55) or serum magnesium (*p* = 0.11, Supplementary Table S4). These findings indicate that concomitant infection is associated with alterations in zinc homeostasis and an increased Cu/Zn ratio, consistent with an inflammatory response, whereas serum copper and magnesium concentrations appear to remain relatively unaffected. Accordingly, concomitant infection should be considered a potential confounding factor when interpreting serum zinc levels and the Cu/Zn ratio in patients with nephrotic syndrome. These infection-related alterations in zinc homeostasis and the Cu/Zn ratio are graphically illustrated in Figure 2.

### 3.5. Dynamic Protein-Adjusted Ratios and Long-Term Clinical Course

To correct for the mechanical confounding effect of systemic hypoproteinemia, trace elements were indexed to total serum proteins (zn/proteins, cu/proteins, mg/proteins).

When adjusted, the significant differences previously observed for crude zinc across disease activity groups completely disappeared (*p* = 0.42), confirming that hypozincemia in NS is a transport defect rather than an absolute nutritional deficiency. Conversely, the cu/proteins (*p* = 0.001) and mg/proteins (*p* < 0.001) ratios remained significantly elevated during active disease states (Table 6).

None of the evaluated trace element parameters correlated significantly with relapse number or annual relapse frequency. Disease duration showed modest associations with serum zinc (rho = 0.37, *p* < 0.001), serum magnesium (rho = −0.22, *p* = 0.01), and fractional magnesium excretion (rho = 0.31, *p* < 0.001) (Supplementary Table S5).

### 3.6. Diagnostic Performance (ROC Curve Analysis)

**Figure 3:**
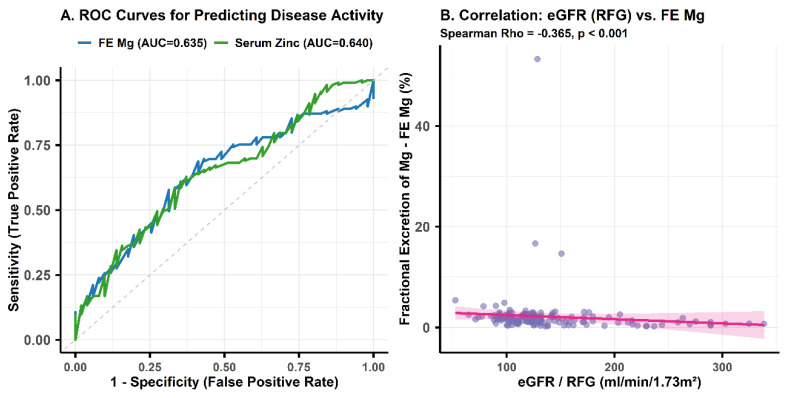
Diagnostic accuracy for disease activity and functional correlation between filtration capacity and magnesium homeostasis. (**A**) Receiver Operating Characteristic (ROC) curves evaluating univariate baseline parameters for predicting active nephrotic states (Active Flare vs. Controls/Remission). The dotted diagonal line represents the reference (no-discrimination) line. Although serum zinc and FEMg achieved statistically significant AUC values, their discriminatory performance was modest (AUC ≈0.64) and unlikely to provide clinically useful information beyond standard clinical assessment. (**B**) Scatterplot with a linear purple fit line and 95% confidence interval shading depicting the negative correlation between estimated Glomerular filtration rate and FEMg (Spearman’s rho = − 0.37, *p* < 0.001), confirming enhanced tubular magnesium conservation during hyperfiltrative active flares.

Receiver Operating Characteristic (ROC) curves were generated to test the diagnostic power of baseline trace elements (Supplementary Table S6). ROC analysis showed only modest discrimination of disease activity for serum zinc (AUC 0.64) and FEMg (AUC 0.64). Neither biomarker provided clinically useful discrimination beyond routine clinical and laboratory assessment. These findings, together with the relationship between renal function and fractional magnesium excretion, are illustrated in Figure 3. Likewise, none of the evaluated trace element parameters predicted steroid resistance in univariable analyses (all AUCs < 0.60).

### 3.7. Multivariable Predictive Regression Models

#### 3.7.1. Independent Predictors of Disease Activity

A multivariable logistic regression model was constructed to isolate independent predictors of active disease status (N = 153 valid entries, Table 7). After forcing microelements, proteinuria, inflammatory markers, and age into the model, total serum proteins emerged as the single, robust independent predictor (*p* < 0.001, Odds Ratio [OR] = 0.80, 95% CI: 0.72–0.90). This indicates that for every 1 g/L increase in total serum proteins, the odds of a patient experiencing an active nephrotic flare decrease independently by approximately 19.8%, overriding the univariable effects of trace elements.

#### 3.7.2. Independent Predictors of Steroid Resistance

**Figure 4:**
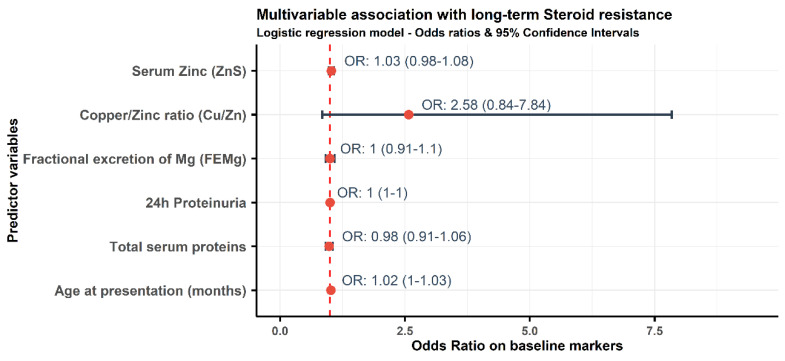
Forest plot of multivariable logistic regression predictors for long-term steroid resistance. Graphical representation of independent baseline clinical and metabolic risks derived from the multivariable logistic model. Dots represent specific Odds Ratios (OR), while horizontal bars denote the 95% Confidence Intervals. Older age at presentation was the only statistically significant independent predictor of steroid resistance (OR = 1.02, 95% CI: 1.01–1.03; *p* = 0.004). The red vertical dashed line indicates the null-effect threshold (OR = 1.0).

A second multivariable logistic regression model was constructed to identify independent baseline predictors of long-term steroid resistance (N = 126). Older age at presentation was the only independent predictor of steroid resistance (OR = 1.02, 95% CI: 1.01–1.03; *p* = 0.004), corresponding to a 1.7% increase in the odds of steroid resistance for each additional month of age at disease onset. None of the evaluated trace element parameters, including serum zinc, the Cu/Zn ratio, and fractional magnesium excretion, remained independently associated with steroid resistance after multivariable adjustment. The multivariable logistic regression model identifying independent predictors of steroid resistance is graphically summarized in Figure 4.

## 4. Discussion

The homeostatic balance of essential trace elements—zinc, copper, and magnesium—is severely disrupted during the active phases of pediatric NS. The results of this prospective study provide clear statistical and physiological indications that these changes are closely linked to the integrity of the glomerular filtration barrier and systemic protein configurations.

**The Zinc–Protein axis and the transport misconception**. Univariable analysis demonstrated marked hypozincemia during active NS (initial presentation and relapse), with progressive normalization during stable remission. However, after adjustment for total serum protein concentration, differences in serum zinc levels between active disease and healthy controls disappeared (*p* = 0.42). Furthermore, multivariable logistic regression identified total serum protein concentration as the only independent predictor of disease activity (*p* < 0.001, OR = 0.80), overriding the apparent association observed for unadjusted serum zinc concentrations. These findings support the concept that hypozincemia in pediatric NS primarily reflects impaired protein-bound zinc transport secondary to hypoproteinemia rather than absolute zinc deficiency [10,36,37]. Consequently, isolated serum zinc concentrations should be interpreted cautiously and do not support routine zinc supplementation based solely on biochemical hypozincemia. Nevertheless, additional alterations in zinc homeostasis related to oxidative stress and inflammatory activation, including metallothionein-mediated redistribution, may further contribute to reduced circulating zinc concentrations [38,39].

**Infectious triggers and syndromic amplification.** Children with concomitant infection had significantly lower serum zinc concentrations (median 35.90 vs. 65.40 μg/dL, *p* < 0.001) and higher Cu/Zn ratios (median 1.90, *p* = 0.001), whereas serum copper and magnesium concentrations remained unchanged. Although the zinc-to-total protein ratio was lower in infected patients (median 0.93 vs. 1.02), the difference did not reach statistical significance (*p* = 0.08). These findings are consistent with the acute-phase response described by Liuzzi et al., in which interleukin-6 induces hepatic expression of the zinc transporter ZIP14, promoting redistribution of zinc from plasma into hepatocytes and resulting in reduced circulating zinc concentrations independently of urinary protein losses [40,41]. Therefore, concomitant infection should be considered an important confounding factor when interpreting serum zinc concentrations in children with NS. Taken together, the present findings suggest that hypozincemia in pediatric NS is predominantly related to reduced protein-bound zinc transport, while inflammatory zinc redistribution may represent an additional mechanism during infectious episodes.

**Hypercupruria as a marker of permeability.** Active NS was characterized by marked hypercupruria, increased copper clearance, and elevated Cu/Zn ratios (all *p* < 0.001). Urinary copper concentration (rho = +0.35) and copper clearance (rho = +0.38) correlated positively with 24-h proteinuria, supporting increased urinary loss of copper-transporting proteins following disruption of the glomerular filtration barrier [8,42,43,44]. Although copper metabolism has been less extensively investigated in pediatric NS and previous studies have reported heterogeneous serum findings [45], increased urinary copper excretion during active disease has been consistently described [14,44,46,47,48]. The present study complements previous findings by incorporating copper clearance measurements and evaluating the Cu/Zn ratio as a marker associated with disease activity, reaching a median value of approximately 1.9 during active disease before normalizing in remission. In the present cohort, the Cu/Zn ratio paralleled disease activity and may reflect alterations in trace element homeostasis and inflammatory status; however, its clinical utility remains to be established in larger prospective pediatric studies [49,50,51,52,53].

**Tubular adaptation and the magnesium paradox.** Although serum magnesium decreased only slightly during active proteinuria, renal magnesium handling demonstrated a marked adaptive response. Twenty-four-hour urinary magnesium excretion, magnesium clearance, and fractional excretion of magnesium (FEMg) reached their lowest values during disease onset (median FEMg 0.70%) and increased significantly during stable remission (2.17%). The inverse correlation between proteinuria and FEMg (rho = −0.39, *p* < 0.001) suggests enhanced tubular magnesium reabsorption, potentially mediated by TRPM6/TRPM7 transporters, as a compensatory mechanism limiting systemic magnesium loss [54,55]. These findings extend the limited literature regarding renal magnesium handling in pediatric NS, which has focused predominantly on serum magnesium despite its limited ability to reflect total body magnesium stores [23,56,57,58,59]. Because calcineurin inhibitors are known to induce renal magnesium wasting through TRPM6 downregulation [2,60], the present observations may provide a physiological reference for distinguishing disease-related tubular adaptation from treatment-induced alterations in magnesium homeostasis.

**Long-term clinical correlations.** Neither serum zinc, serum copper, serum magnesium nor FEMg were associated with cumulative relapse number or annual relapse frequency. However, disease duration correlated positively with serum zinc concentrations (rho = 0.37, *p* < 0.001) and FEMg (rho = 0.31, *p* < 0.001), whereas serum magnesium showed a weak inverse correlation (rho = −0.22, *p* = 0.01). Because previous studies have primarily compared active disease with remission, these observations should be regarded as exploratory. They suggest that trace element homeostasis may continue to evolve throughout the chronic course of pediatric NS, although prospective longitudinal studies are required to clarify the underlying mechanisms and their clinical significance.

**Predictive models and limitations.** Univariable ROC analysis demonstrated only moderate discrimination of active disease for serum zinc (AUC = 0.64) and FEMg (AUC = 0.64). Neither biomarker independently predicted steroid resistance. In multivariable logistic regression, older age at disease onset emerged as the only independent predictor of steroid resistance (*p* = 0.004, OR = 1.02), consistent with previous pediatric cohorts reporting an increased risk of steroid resistance and focal segmental glomerulosclerosis among older children at presentation [1,2,3,61,62,63]. These findings indicate that the evaluated trace element biomarkers do not provide predictive value beyond established clinical predictors in the present cohort.

The main limitation of this study is the relatively small number of patients with histopathological confirmation (N = 29), a consequence of current recommendations regarding restrictive indications for renal biopsy in the pediatric population. This aspect limited the ability to analyze in depth the differences in trace element homeostasis between histological variants of NS, such as minimal change disease and focal segmental glomerulosclerosis. Furthermore, the study was conducted at a single tertiary center, which may limit the generalizability of the results to other pediatric populations. Additional limitations include the lack of urinary zinc measurements and the absence of free (unbound) zinc, copper, and magnesium determinations, which prevented a direct evaluation of urinary zinc losses and biologically active circulating fractions. Furthermore, because several patients contributed repeated observations obtained during different stages of disease evolution, the analyses should be interpreted as exploratory. Finally, no formal sample size calculation or statistical adjustment for multiple comparisons was performed; therefore, the findings should be interpreted as hypothesis-generating and require confirmation in larger prospective studies. Although the multivariable models controlled for key clinical and biological confounders and no relevant multicollinearity was identified among the included predictors, the molecular mechanisms involved in trace element homeostasis—such as levels of ceruloplasmin or metallothioneins, or the expression of tubular magnesium transporters (TRPM6/TRPM7)—were not evaluated; thus, experimental studies are needed to confirm the underlying mechanisms. Finally, the relatively small number of steroid-resistant patients reduced the statistical power of the predictive analyses and may have limited the detection of weaker associations.

## 5. Conclusions

Pediatric idiopathic nephrotic syndrome is associated with consistent, stage-dependent alterations in zinc, copper, and magnesium homeostasis that are closely linked to the degree of proteinuria and disruption of the glomerular filtration barrier, reflecting integrated systemic and renal adaptive responses rather than isolated biochemical abnormalities. Our findings indicate that low serum zinc levels during active disease are predominantly proteinuria-dependent, whereas copper handling more directly reflects glomerular permeability through increased urinary excretion and clearance, and magnesium exhibits a reversible pattern of tubular conservation during active disease. These observations highlight the importance of interpreting trace element status within the context of protein loss and renal handling rather than relying on isolated serum measurements. Clinically, the Cu/Zn ratio and fractional magnesium excretion emerged as complementary indicators of disease activity and renal adaptation, while total serum protein remained the strongest independent determinant of disease activity; trace element-derived indices may provide additional value for clinical phenotyping and potential steroid response prediction. Finally, the association of several trace element parameters with disease duration, rather than cumulative relapse burden, suggests that chronic disease evolution may influence micronutrient homeostasis independently of acute disease activity. Larger prospective multicenter studies are warranted to validate these findings and establish their clinical utility.

## Figures and Tables

**Table 1:** Demographic and anthropometric characteristics of the participants, by study group.

Characteristic	Control (N = 34)	New-Onset NS (N = 30)	Chronic Active NS (N = 83)	Chronic Inactive NS (N = 17)
Age (months), Median (IQR)	101.5 (60.8–169.2)	41.5 (31.2–63.5)	91 (57.5–138.5)	122 (86–148)
Gender (Male), N (%)	20 (58.8%)	24 (80%)	50 (60.2%)	10 (58.8%)
Weight (kg), Mean ± SD	36.83 ± 18.63	20.97 ± 9.88	32.55 ± 14.97	42.41 ± 19.34
Height (cm), Mean ± SD	136.85 ± 26.76	105.6 ± 16.78	127.6 ± 22.63	142.71 ± 21.59
BMI (kg/m^2^), Mean ± SD	18.21 ± 3.73	18.18 ± 2.93	18.98 ± 3.79	19.89 ± 5.75

**Table 2:** Clinical, evolutionary, and therapeutic characteristics of patients with nephrotic syndrome according to disease stage.

Characteristic	New-Onset NS (N = 30)	Chronic Active NS (N = 83)	Chronic Inactive NS (N = 17)
**Episode Type, N (%):**			
Initial presentation	11 (36.7%)	0 (0%)	0 (0%)
Relapse	4 (13.3%)	24 (28.9%)	0 (0%)
Initial remission	9 (30%)	0 (0%)	0 (0%)
Early remission	2 (6.7%)	20 (24.1%)	0 (0%)
Late remission	4 (13.3%)	39 (47%)	0 (0%)
Sustained remission	0 (0%)	0 (0%)	17 (100%)
*Disease duration* (months), Mean ± SD	2.57 ± 2.91	45.52 ± 47.04	76.94 ± 39.02
Number of relapses, Mean ± SD	0.23 ± 0.43	3.64 ± 3.63	3.24 ± 2.91
Relapse frequency (per year), Mean ± SD	0.55 ± 1.09	1.31 ± 0.89	0.49 ± 0.4
**Steroid Response, N (%):**			
Steroid-dependent	9 (30%)	24 (28.9%)	5 (29.4%)
Steroid-resistant	2 (6.7%)	18 (21.7%)	5 (29.4%)
Steroid-sensitive	19 (63.3%)	41 (49.4%)	7 (41.2%)
Nephrotic Syndrome Type *, N (%):			
Pure	25 (83.3%)	73 (88%)	13 (76.5%)
Impure	5 (16.7%)	10 (12%)	4 (23.5%)
**Concomitant Infection**: Yes, N (%)	6 (20%)	6 (7.2%)	1 (5.9%)
**Treatment, N (%):**			
None	12 (40%)	28 (33.7%)	16 (94.1%)
Prednisone	8 (26.7%)	12 (14.5%)	0 (0%)
Prednisone alternative	7 (23.3%)	18 (21.7%)	0 (0%)
Ciclosporin	1 (3.3%)	11 (13.3%)	1 (5.9%)
Mycophenolate	0 (0%)	1 (1.2%)	0 (0%)
Ciclosporin & Prednisone	2 (6.7%)	6 (7.2%)	0 (0%)
Cyclophosphamide & Prednisone	0 (0%)	4 (4.8%)	0 (0%)
Mycophenolate & Prednisone	0 (0%)	1 (1.2%)	0 (0%)
Ciclosporin & Mycophenolate	0 (0%)	2 (2.4%)	0 (0%)

* Note: Pure nephrotic syndrome was defined by the absence of hematuria, hypertension, impaired kidney function, and hypocomplementemia. Impure nephrotic syndrome was defined by the presence of one or more of these features.

**Table 3:** Distribution of laboratory parameters according to the type of clinical episode in patients with nephrotic syndrome—statistically significant data.

	Initial Presentation	Relapse	Initial Remission	Early Remission	Late Remission	Sustained Remission	*p*-Value Kruskal–Wallis
***Biochemistry***							
Total Proteins (g/L)	36.4 (33.8–40.45)	48.35 (44.17–54.85)	63.6 (61.2–67.7)	67.25 (62.52–68.75)	67 (64–68.9)	71.8 (70–75.9)	**<0.001**
Albumin (g/L)1	10.4 (6.62–12)	22.47 (9.31–29.56)	35.88 (35.88–35.88)	44.72 (44.72–44.72)	N/A	36.95 (36.09–37.82)	**0.02**
Creatinine (mg/dL)	0.16 (0.15–0.23)	0.29 (0.21–0.4)	0.35 (0.25–0.39)	0.45 (0.36–0.52)	0.42 (0.34–0.48)	0.46 (0.4–0.58)	**<0.001**
Total Cholesterol (mg/dL)	482.5 (439.5–564)	287.5 (204.5–363.75)	195 (170–257)	211 (188.75–231.5)	166 (147–182)	170.5 (142.25–176.25)	**<0.001**
Triglycerides (mg/dL)	280 (188–459)	122 (88–180.5)	100 (78–143)	100 (86.75–125.75)	73 (56.75–102)	59.5 (52–75.25)	**<0.001**
ESR	55.5 (34.25–65.75)	24.5 (17.75–46.25)	6 (2–10)	5.5 (3.5–9.25)	9.5 (4–11)	6.5 (6–16.75)	**<0.001**
eGFR (mL/min/1.73 m^2^)	229.73 (166.16–272.58)	171.02 (146.76–217.94)	123.9 (118–161.9)	110.52 (101.67–127.81)	122.98 (103.49–131.91)	124.93 (113.58–131.08)	**<0.001**
***Urine***							
24-h proteinuria (mg/24-h)	3761.88 (1855.78–5208.35)	3704.48 (2190–5423.45)	124.08 (91.97–134.3)	131.43 (97.05–175.42)	91.58 (64.43–127.85)	136 (72–198.9)	**<0.001**
***Microelements***							
Serum zinc	32.1 (28.35–43.35)	48 (37.17–62.02)	60.8 (59.3–67)	76.4 (65.17–87.85)	68 (60.25–76.35)	72.2 (66–79.7)	**<0.001**
Serum copper	61.9 (50.9–84.6)	96.45 (80.22–103.88)	74.7 (69.6–78.7)	76.75 (63.1–92.5)	96.6 (84.25–105.15)	88.4 (79.1–111.7)	**<0.001**
Copper/zinc ratio	1.9 (1.33–2.26)	1.85 (1.39–2.61)	1.25 (1.08–1.33)	1.02 (0.84–1.15)	1.43 (1.12–1.69)	1.24 (1.11–1.58)	**<0.001**
Serum magnesium	2.07 (1.96–2.16)	1.85 (1.76–1.97)	2.08 (1.99–2.25)	2.11 (1.92–2.24)	1.86 (1.7–2.06)	1.92 (1.83–2)	**0.006**
24-h Urinary copper excretion	73.35 (48.88–145.42)	84.22 (36.66–120.73)	0 (0–9.36)	14.4 (0–52.25)	1.35 (0–28.54)	0 (0–24)	**<0.001**
24-h Urinary magnesium excretion	20.6 (6.56–44.2)	32.49 (19.18–55.22)	16.32 (11.98–27.66)	38.15 (28.36–61.52)	50.39 (33.26–66.66)	55 (47.6–90)	**<0.001**
Fractional excretion of magnesium	0.7 (0.3–1.07)	1.06 (0.58–1.94)	1.07 (0.74–1.28)	1.39 (0.77–1.95)	1.88 (1.35–2.52)	2.17 (1.94–2.41)	**<0.001**

**Note**: Bold values indicate statistically significant results (*p* < 0.05).

**Table 4:** The correlation between trace element metabolism parameters and proteinuria, evaluated using the Spearman correlation coefficient.

Parameter	Spearman’s Correlation Coefficient (ρ)	*p*-Value
Serum zinc	−0.38	**<0.001**
Serum copper	−0.13	0.09
Copper/zinc ratio	0.23	**0.003**
Serum magnesium	−0.08	0.30
Urinary copper	0.35	**<0.001**
Copper clearance	0.38	**<0.001**
Urinary magnesium	−0.11	0.16
Magnesium clearance	0.04	0.61
Fractional excretion of magnesium	−0.39	**<0.001**

**Note**: Bold values indicate statistically significant results (*p* < 0.05).

**Table 5:** Spearman correlation analyses of trace element parameters with inflammatory markers.

Parameter/Comparison	Spearman’s Correlation Coefficient (ρ)	*p*-Value
**Correlation with inflammatory markers**		
Serum zinc ↔ CRP	−0.12	0.14
Serum zinc ↔ ESR	−0.32	**0.001**
Serum copper ↔ CRP	0.23	**0.004**
Serum copper ↔ ESR	0.18	0.07
Copper/zinc ratio ↔ CRP	0.25	**0.002**
Copper/zinc ratio ↔ ESR	0.44	**<0.001**

**Note**: Bold values indicate statistically significant results (*p* < 0.05).

**Table 6:** Comparison of serum trace element to total protein ratios among study groups.

Ratio Parameter	New-Onset NS (Median, IQR)	Chronic Active NS (Median, IQR)	Chronic Inactive NS (Median, IQR)	Control (Median, IQR)	Kruskal–Wallis *p*-Value
Zinc/total protein ratio	0.97 (0.82–1.14)	1.02 (0.90–1.18)	0.98 (0.91–1.12)	0.96 (0.780–1.10)	0.42
Copper/total protein ratio	1.50 (1.17–1.79)	1.47 (1.21–1.76)	1.32 (1.21–1.45)	1.178 (0.98–1.36)	**0.001**
Magnesium/total protein ratio	0.035 (0.033–0.054)	0.031 (0.027–0.035)	0.026 (0.025–0.029)	0.028 (0.027–0.030)	**<0.001**

**Note**: Bold values indicate statistically significant results (*p* < 0.05).

**Table 7:** Multivariable logistic regression analysis of predictors of disease activity.

Predictor Variable	Odds Ratio (OR)	95% CI	*p*-Value
Serum zinc	1.01	0.97–1.04	0.79
Copper/zinc ratio	1.03	0.22–4.77	0.97
Fractional excretion of magnesium	1.13	0.83–1.55	0.44
Proteinuria	1.002	1.00–1.01	0.36
Serum total proteins	0.80	0.72–0.90	**<0.001**
C-reactive protein (CRP)	0.88	0.78–1.00	0.06
Age (months)	1.00	0.99–1.01	0.53

**Note**: Bold values indicate statistically significant results (*p* < 0.05).

## Data Availability

Data is available on request from the corresponding author due to ethical issues.

## Supplementary Materials

The following supporting information can be downloaded at: https://www.mdpi.com/article/10.3390/nu18152529/s1, Supplementary Table S1. Distribution of laboratory parameters according to the type of clinical episode in patients with nephrotic syndrome - additional correlations. Supplementary Table S2. Spearman correlation analysis between trace element parameters and total serum proteins. Supplementary Table S3. Spearman correlation analyses of trace element parameters with eGFR. Supplementary Table S4. Comparison of serum trace element parameters according to the presence of concomitant infection. Supplementary Table S5. Spearman correlation between trace element parameters and clinical indicators of disease activity. Supplementary Table S6. Receiver operating characteristic (ROC) analysis of trace element biomarkers for predicting disease activity and steroid resistance.
