# Supplementary material for: Integrated Analysis of Zinc, Copper, and Magnesium Homeostasis in Pediatric Idiopathic Nephrotic Syndrome: A Prospective Cohort Study with Serial Clinical Evaluation
Source: Nutrients. 2026 Aug 4;18(15):2529. doi: 10.3390/nu18152529 (PMC13467790; doi:10.3390/nu18152529)
Supplement: Supplementary file 1 [file nutrients-18-02529-s001.zip › Supplementary Table.pdf]

Supplementary Table S1. Distribution of laboratory parameters according to the type of clinical episode in patients with nephrotic syndrome - additional correlations.

	Initial presentation	Relapse	Initial remission	Early remission	Late remission	Sustained remission	p-value Kruskal-Wallis
Hematology							
Hb (g/dL)	12.9 (11.9 - 13.8)	13.25 (12.3 - 13.9)	13.4 (12.2 - 13.8)	13.4 (12.88 - 14.25)	12.9 (12.25 - 13.4)	13.3 (12.8 - 14)	0.20
Hct (%)	37.8 (34.3 - 38.7)	38.4 (36.3 - 40.05)	38.9 (37.2 - 39.9)	40.45 (38.35 - 42.1)	37.6 (35.75 - 39.4)	39.3 (37.3 - 40.3)	**0.04**
VEM (fL)	77.1 (74.5 - 80.3)	76.4 (73.95 - 79)	78.6 (75.9 - 79)	78.8 (75 - 80.65)	78.6 (76.25 - 81.25)	80.7 (76.8 - 83.3)	0.09
CHEM (g/dL)	34.7 (34 - 35.35)	34.7 (34.03 - 35.1)	33.9 (33 - 34.6)	33.7 (32.7 - 34.48)	34.4 (33.65 - 34.8)	34.3 (33.9 - 35.3)	0.09
HEM (pg)	26.9 (25.8 - 28.15)	26.25 (25.6 - 27.52)	27.3 (25 - 27.8)	26.45 (25.12 - 27.45)	27.1 (26 - 28.25)	27.7 (27.1 - 28.6)	0.22
Biochemistry							
Urea (mg/dL)	30 (24.5 - 49.5)	26 (21.75 - 31)	23 (23 - 34)	31 (27 - 37)	26 (21 - 33.5)	26 (23 - 30)	0.20
Sodium - Na (mmol/L)	139 (137 - 141)	139 (137 - 140)	139 (137.5 - 140.5)	142 (142 - 142)	138.5 (138 - 139.75)	139.5 (139.25 - 139.75)	0.73
Potassium - K (mmol/L)	4.34 (4.21 - 4.55)	4.38 (4.3 - 4.5)	4.7 (4.45 - 4.83)	4.2 (4.2 - 4.2)	4.08 (3.88 - 4.42)	4.44 (4.15 - 4.74)	0.73
Calcium - Ca (mg/dL)	4.65 (4.45 - 4.93)	4.66 (4.49 - 4.75)	4.85 (4.67 - 5.03)	4.74 (4.58 - 4.78)	4.61 (4.41 - 4.77)	4.43 (4.23 - 4.64)	0.35
Phosphorus - P (mg/dL)	5.1 (4.35 - 5.85)	5.3 (5.05 - 5.8)	5 (4.8 - 5.2)	4.3 (4.2 - 4.95)	4.3 (4.1 - 5.35)	4.6 (4.35 - 4.65)	0.08
LDL Cholesterol (mg/dL)	438 (438 - 438)	260 (260 - 260)	N/A	165 (165 - 165)	82 (82 - 82)	86 (86 - 86)	-
CRP (mg/L)	0.5 (0.5 - 1.7)	0.5 (0.5 - 1.55)	0.5 (0.5 - 0.5)	0.5 (0.22 - 1.3)	0.5 (0.5 - 0.9)	0.5 (0.5 - 1.3)	0.63
Urine							
24h urine volume (mL/24h)	540 (372.5 - 1112.5)	740 (495 - 1550)	880 (655 - 1000)	1087.5 (892.5 - 1600)	1125 (762.5 - 1500)	1200 (900 - 1500)	**0.02**
Urine creatinine (mg/dL)	293.37 (259.6 - 351.9)	450.82 (308.02 - 693.21)	294.4 (185.39 - 518)	584.49 (434.49 - 819.61)	537.71 (400.5 - 740.74)	819.9 (541.88 - 1160.04)	**0.001**
Microelements							
Urine copper concentration	13.75 (6.23 - 31.17)	8.65 (4.35 - 15.97)	0 (0 - 1.75)	0.95 (0 - 4.4)	0.15 (0 - 3.4)	0 (0 - 1.6)	**< 0.001**
Copper clearance	0.06 (0.05 - 0.12)	0.06 (0.03 - 0.09)	0 (0 - 0.01)	0.01 (0 - 0.04)	0 (0 - 0.02)	0 (0 - 0.01)	**< 0.001**

Urine magnesium concentration	3.77 (2.82 - 4.46)	3.4 (2.06 - 6.32)	1.46 (1.23 - 3.1)	3.06 (2.03 - 6.69)	4.65 (2.62 - 7.14)	4.55 (3.97 - 6.37)	**0.05**
Magnesium clearance	0.81 (0.23 - 1.5)	1.19 (0.66 - 2.02)	0.61 (0.46 - 0.95)	1.17 (0.94 - 1.99)	1.81 (1.21 - 2.47)	2.15 (1.65 - 3.22)	**< 0.001**

Supplementary Table S2. Spearman correlation analysis between trace element parameters and total serum proteins

**Parameter**	**Spearman's correlation coefficient (ρ)**	***p*-value**	***N***
Serum zinc	0.59	**< 0.001**	164
Serum copper	0.13	0.11	164
Serum magnesium	0.16	**0.04**	164
Copper/zinc ratio	-0.38	**< 0.001**	164

Supplementary Table S3. Spearman correlation analyses of trace element parameters with eGFR

**Parameter / Comparison**	**Spearman's correlation coefficient (ρ)**	***p*-value**
**Correlation with eGFR**		
Serum zinc	-0.40	**< 0.001**
Serum copper	0.02	0.79
Serum magnesium	-0.19	**0.02**
Magnesium clearance	-0.14	0.08
Fractional excretion of magnesium	-0.37	**< 0.001**

Supplementary Table S4. Comparison of serum trace element parameters according to the presence of concomitant infection

**Parameter**	**Infection Present (Median, IQR)**	**No Infection (Median, IQR)**	***p*-value**
Serum zinc	35.9 (30.6–45.4)	65.4 (54.0–76.2)	**< 0.001**
Serum copper	88.4 (63.1–97.3)	86.1 (75.2–102.7)	0.55
Copper/zinc ratio	1.90 (1.46–2.84)	1.31 (1.07–1.68)	**0.001**
Serum magnesium	2.07 (1.89–2.23)	1.92 (1.76–2.07)	0.12

Supplementary Table S5. Spearman correlation between trace element parameters and clinical indicators of disease activity

**Element Parameter**	**Clinical Metric**	**Spearman's correlation coefficient (ρ)**	***p*-value**
Serum zinc	Number of Relapses	0.15	0.09
	Relapse Frequency/Year	-0.06	0.53
	Disease Duration (months)	0.37	**< 0.001**
Serum copper	Number of Relapses	0.14	0.10
	Relapse Frequency/Year	0.11	0.20
	Disease Duration (months)	0.16	0.08
Serum magnesium	Number of Relapses	-0.15	0.08
	Relapse Frequency/Year	-0.03	0.70
	Disease Duration (months)	-0.22	**0.01**
Fractional excretion of magnesium	Number of Relapses	0.14	0.12
	Relapse Frequency/Year	-0.06	0.51
	Disease Duration (months)	0.31	**< 0.001**

Supplementary Table S6. Receiver operating characteristic (ROC) analysis of trace element biomarkers for predicting disease activity and steroid resistance

**Clinical Outcome**	**Biomarker**	**AUC (95% CI)***	**Cut-off**	**Sensitivity**	**Specificity**	***p*-value**
**Disease Activity**	Serum zinc	0.64	65.85	61.1%	64.7%	**0.004**
	Copper/zinc ratio	0.59	1.00	86.7%	31.4%	0.08
	Fractional excretion of magnesium	0.64	1.90	68.8%	58.8%	0.006
**Steroid Resistance**	Serum zinc	0.54	76.10	40.0%	80.0%	0.50
	Copper/zinc ratio	0.52	1.40	60.0%	54.3%	0.71
	Fractional excretion of magnesium	0.57	1.34	72.0%	47.5%	0.28
